# Suppressing IL-36-driven inflammation using peptide pseudosubstrates for neutrophil proteases

**DOI:** 10.1038/s41419-018-0385-4

**Published:** 2018-03-07

**Authors:** Graeme P. Sullivan, Conor M. Henry, Danielle M. Clancy, Tazhir Mametnabiev, Ekaterina Belotcerkovskaya, Pavel Davidovich, Sylvia Sura-Trueba, Alexander V. Garabadzhiu, Seamus J. Martin

**Affiliations:** 1 Molecular Cell Biology Laboratory, Department of Genetics, The Smurfit Institute, Trinity College, Dublin 2, Ireland; 2 Cellular Biotechnology Laboratory, Saint-Petersburg Technical University, Moskovskii Prospekt, Saint Petersburg, Russia

## Abstract

Sterile inflammation is initiated by molecules released from necrotic cells, called damage-associated molecular patterns (DAMPs). Members of the extended IL-1 cytokine family are important DAMPs, are typically only released through necrosis, and require limited proteolytic processing for activation. The IL-1 family cytokines, IL-36α, IL-36β, and IL-36γ, are expressed as inactive precursors and have been implicated as key initiators of psoriatic-type skin inflammation. We have recently found that IL-36 family cytokines are proteolytically processed and activated by the neutrophil granule-derived proteases, elastase, and cathepsin G. Inhibitors of IL-36 processing may therefore have utility as anti-inflammatory agents through suppressing activation of the latter cytokines. We have identified peptide-based pseudosubstrates for cathepsin G and elastase, based on optimal substrate cleavage motifs, which can antagonize activation of all three IL-36 family cytokines by the latter proteases. Human psoriatic skin plaques displayed elevated IL-36β processing activity that could be antagonized by peptide pseudosubstrates specific for cathepsin G. Thus, antagonists of neutrophil-derived proteases may have therapeutic potential for blocking activation of IL-36 family cytokines in inflammatory conditions such as psoriasis.

## Introduction

IL-1 family cytokines play major roles as initiators of inflammation, are typically only released upon necrotic injury, and are likely to represent canonical danger-associated molecular patterns (DAMPs)^1–4^. IL-1 family cytokines act on multiple cell types, such as macrophages, dendritic cells, keratinocytes, and endothelial cells lining local blood vessels^5–10^. IL-36α, β, and γ are recently described members of the IL-1 family and exhibit many of the characteristic features of IL-1 family cytokines, including the requirement for N-terminal processing to release their full biological activity. As we and others have demonstrated, removal of just a small number of residues from the N termini of IL-36 cytokines radically increases their biological activity^11–13^. This tight regulatory control on their activity probably represents a mechanism to limit the potential negative consequences if the activity of these cytokines is deregulated.

IL-36α, IL-36β, and IL-36γ, which are encoded by distinct genes, are non-conventionally secreted and it is now well established that these cytokines are important modulators of inflammation in barrier tissues, particularly in skin inflammatory diseases such as psoriasis^14–24^. Partial loss-of-function mutations in the IL-36 receptor antagonist (IL-36RA) can lead to a highly debilitating morbid form of psoriasis, termed generalized pustular psoriasis^17,18,21–23^. Furthermore, analysis of skin biopsies from individuals with the most common form of psoriasis, psoriasis vulgaris, shows significantly increased expression (100-fold) of all three IL-36 mRNA transcripts compared with non-lesional skin from the same individuals, or non-affected controls^15,16^. Indeed multiple lines of evidence in vitro and in vivo confirm that deregulated IL-36 cytokine signaling is sufficient to drive aggressive skin inflammation^15–19,25^.

We have recently found that the neutrophil-derived proteases, cathepsin G and elastase, are potent IL-36-activating enzymes^12^. Because psoriasis plaques are frequently associated with neutrophil infiltrates^26–28^, these suggest that targeted inhibition of neutrophil granule proteases may have significant therapeutic potential as inhibitors of IL-36 activation in psoriasis, as well as other inflammatory conditions characterized by neutrophil infiltration. IL-36 cytokine or receptor neutralizing antibody approaches are under development and are progressing to clinical trials^24,29^. While systemic antibody-based cytokine neutralization strategies targeting IL-1, IL-17, and IL-17/23 have greatly improved therapeutic outcomes for patients with severe plaque psoriasis, such therapies are costly and can be associated with serious side effects^30,31^. Targeted, localized inhibition of IL-36 cytokine activation in the skin, through direct application of antagonists of IL-36 proteolytic processing, may be an attractive and cost-effective alternative to systemic cytokine neutralization approaches.

Here, we have identified peptide-based antagonists of IL-36 activation based upon optimal cleavage motifs and substrate preferences for the neutrophil granule proteases, elastase, and cathepsin G. These pseudosubstrates exhibit considerable potency against processing and activation of all three IL-36 cytokines in vitro. We also demonstrate that extracts from human psoriatic skin plaques display elevated IL-36β processing that can be antagonized by the latter inhibitors. Direct application of antagonists of IL-36 processing and activation to inflammatory skin lesions may represent a novel strategy to attenuate psoriatic inflammation.

## Results

### Neutrophil proteases process and activate IL-36 family cytokines

**Fig. 1:**
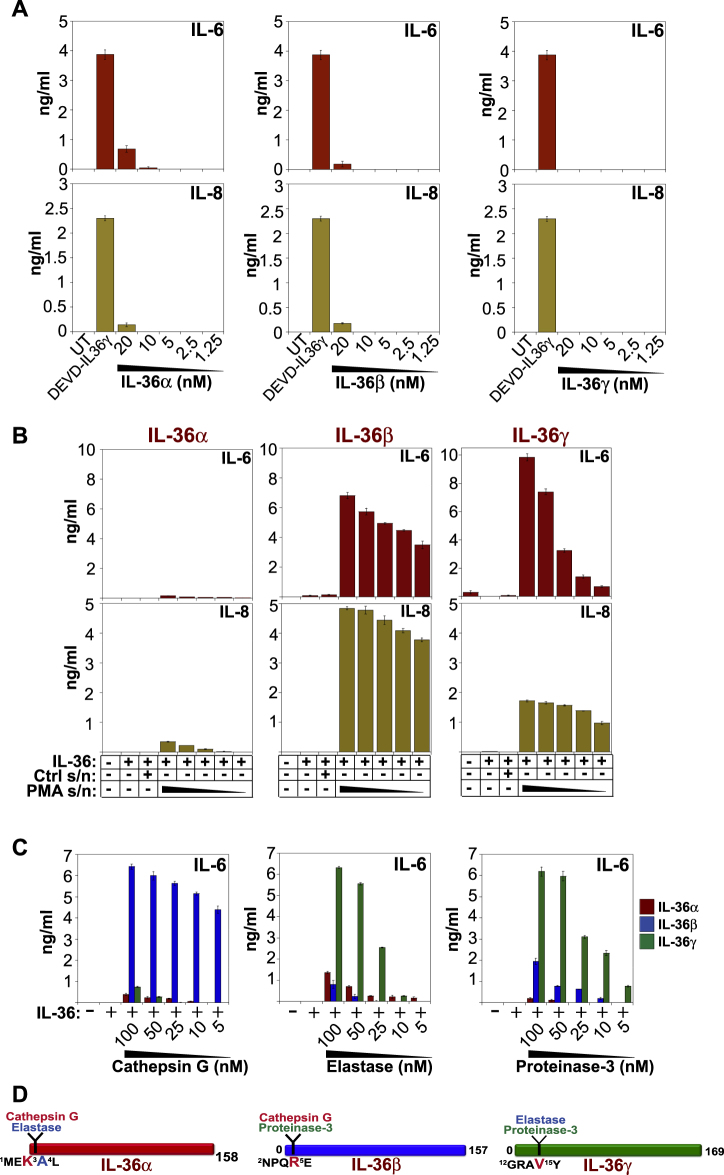
IL-36 cytokines require proteolytic processing for activation. **a** HeLa^IL-36R^ cells were either untreated, or were stimulated with full-length IL-36α, IL-36β, or IL-36γ, at the indicated concentrations. After 24 h, cytokine concentrations in the culture supernatants were determined by ELISA. Caspase-3-cleaved DEVD-IL-36γ (0.5 nM), where a caspase-3-processing motif (DEVD) was inserted into the IL-36 sequence N-terminal to the known processing sites^12,36^, was used as a positive control. **b** HeLa^IL-36R^ cells were stimulated either alone, or in the presence of either IL36α, β, or γ (500 pM) that had been pre-incubated for 2 h at 37 °C with unstimulated neutrophil supernatant (Ctrl s/n), or 1:2 serial dilutions of PMA-activated neutrophil supernatants (PMA s/n). After 24 h, cytokine concentrations in the culture supernatants were determined by ELISA. **c** HeLa^IL-36R^ cells were stimulated either alone, or with IL36α, β, or γ (500 pM) that had been pre-incubated for 2 h at 37 °C with the indicated concentrations of purified human cathepsin G, elastase, or proteinase-3. After 24 h, cytokine concentrations in the culture supernatants were determined by ELISA. **d** Schematic representation of cathepsin G, elastase, and proteinase-3 cleavage motifs within IL-36 family cytokines. Results shown are representative of at least three independent experiments. Error bars represent the mean ±SEM of triplicate determinations from a representative experiment

Similar to other members of the IL-1 family, such as IL-1β and IL-18, IL-36 cytokines exhibit little pro-inflammatory activity as full-length proteins upon incubation with HeLa cells stably transfected with the IL-36 receptor (Fig. 1a). However, as we have recently reported^12^, IL-36 cytokines acquire potent pro-inflammatory activity upon incubation with supernatants derived from PMA-activated human neutrophils that contain the granule-derived proteases, elastase, proteinase-3, and cathepsin G. As shown in Fig. 1b, HeLa^IL-36R^ cells secreted robust amounts of IL-6 and IL-8 upon incubation with recombinant IL-36 cytokines that had been pre-incubated with PMA-activated human neutrophil degranulates, which leads to processing and activation of the latter cytokines^12^. Moreover, incubation of IL-36 cytokines with purified elastase or cathepsin G also robustly activated the latter cytokines, with cathepsin G selectively activating IL-36β, elastase selectively activating IL-36γ, and elastase or cathepsin G both capable of activating IL-36α (Fig. 1c). Figure 1d summarizes the preferences of neutrophil proteases for processing and activation of IL-36 family cytokines and the relevant protease cleavage sites, as recently reported^12^. Thus, activated neutrophils can liberate proteases that can promote inflammation through extracellular processing and activation of IL-36 cytokines.

### Human neutrophil degranulate preparations contain elastase and cathepsin G

**Fig. 2:**
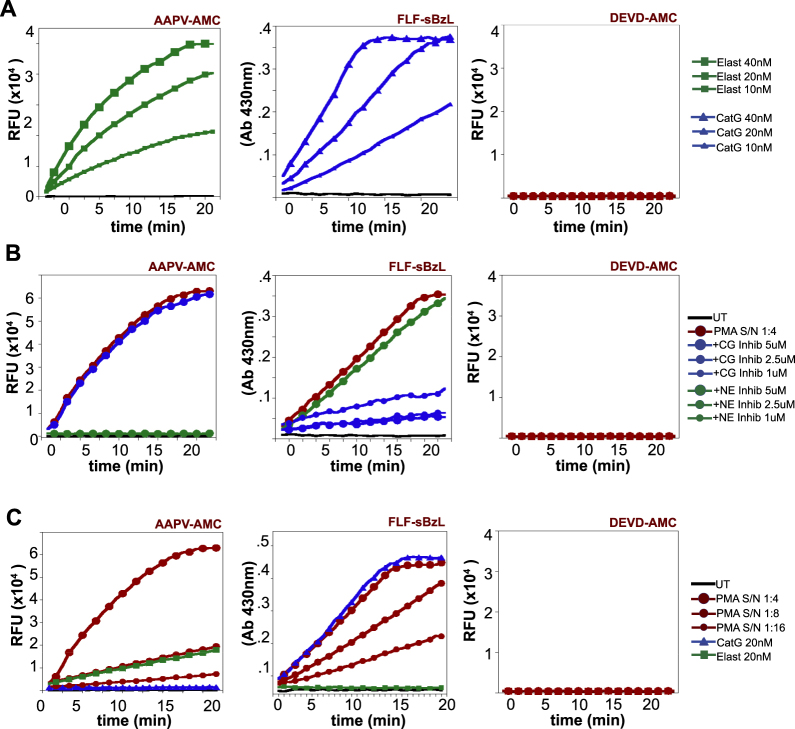
Activated human neutrophils release cathepsin G and elastase. **a** Hydrolysis of synthetic peptide substrates (AAPV-AMC, FLF-sBzl, and DEVD-AMC) by the indicated concentrations of purified elastase, or cathepsin G, as indicated. **b** Hydrolysis of synthetic peptide substrates (AAPV-AMC, FLF-sBzl, and DEVD-AMC) by PMA-activated human neutrophil degranulate (PMA s/n) in the presence or absence of the indicated concentrations of commercially available cathepsin G (CG) or neutrophil elastase (NE) inhibitors. **c** Hydrolysis of synthetic peptide substrates (AAPV-AMC, FLF-sBzl, and DEVD-AMC) by a titration of PMA-activated human neutrophil degranulate, or the indicated amounts of purified cathepsin G or elastase. Results shown are representative of at least three independent experiments

It is well established that primary human neutrophils can be induced to release the contents of their secretory granules into the extracellular space upon activation^32–34^. To establish sensitive assays for the detection of elastase and Cat G activity (Fig. 2a), we used the synthetic substrate peptides, FLF-sBzL (which is cleaved by cathepsin G) and AAPV-AMC (which is cleaved by elastase). Using these synthetic substrates, robust cathepsin G (i.e., FLF-sBzL hydrolysis) and elastase activity (i.e., AAPV-AMC hydrolysis) were readily detected in PMA-activated human neutrophil degranulate preparations, as expected (Fig. 2b). Furthermore, the latter proteases were robustly inhibited by commercially available small molecule inhibitors of cathepsin G, or elastase, respectively (Fig. 2b). Using known amounts of purified cathepsin G or elastase as calibrants (Fig. 2c), we estimated the concentration of the latter enzymes within neutrophil degranulate preparations prepared at 1 × 10^7^ cells/mL to be in the range of ~100–200 nM, respectively.

### Identification of novel peptide-based antagonists of elastase and cathepsin G

**Fig. 3:**
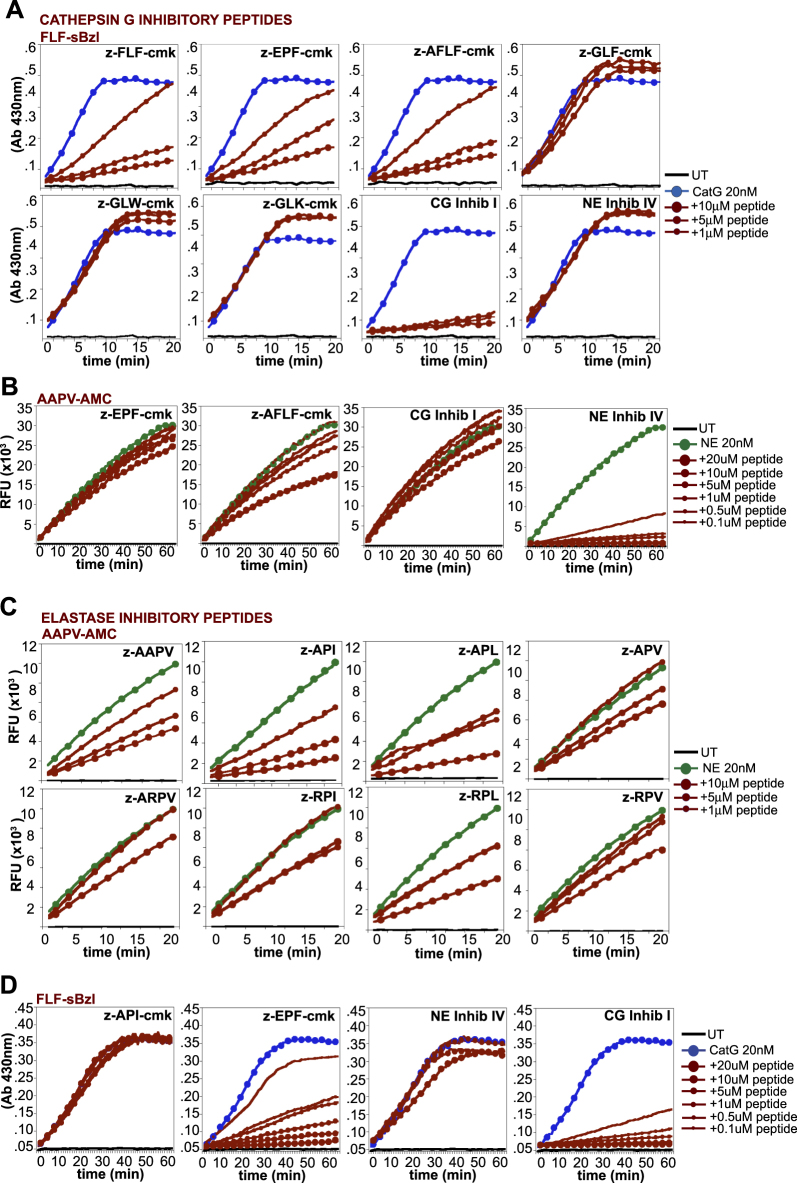
Identification of novel peptide-based inhibitors of cathepsin G and elastase. **a** Hydrolysis of the synthetic cathepsin G substrate, FLF-sBzl, by a fixed concentration of purified cathepsin G (20 nM), in the presence or absence of the indicated concentrations of the candidate cathepsin G peptide inhibitors. **b** Hydrolysis of the synthetic elastase substrate, AAPV-AMC, by a fixed concentration of purified elastase (20 nM), in the presence or absence of the indicated concentrations of the candidate cathepsin G peptide inhibitors. **c** Hydrolysis of the synthetic elastase substrate, AAPV-AMC, by a fixed concentration of purified elastase (20 nM), in the presence or absence of the indicated concentrations of the candidate elastase peptide inhibitors. **d** Hydrolysis of the synthetic cathepsin G substrate, FLF-sBzl, by a fixed concentration of purified cathepsin G (20 nM), in the presence or absence of the indicated concentrations of peptide inhibitors. Cathepsin G inhibitor I (CG Inhib I) or elastase inhibitor IV (NE Inhib IV) served as controls. Results shown are representative of at least three independent experiments

To seek novel peptide-based inhibitors of cathepsin G- and elastase-mediated IL-36 processing and activation, we designed a panel of tri- and tetra-peptides (Table 1), based upon optimal recognition sequences for these proteases^35^, as well as protease cleavage sites implicated in the activation of IL-36 cytokines^12^. We then explored the potential inhibitory activity of this peptide panel toward cathepsin G and elastase. As Fig. 3a demonstrates, several of the candidate cathepsin G inhibitory peptides exhibited robust activity against purified cathepsin G, with z-FLF-cmk, z-EPF-cmk, and z-AFLF-cmk demonstrating the greatest potency in this regard. As expected, none of the latter peptides inhibited elastase activity (Fig. 3b). Similarly, several of the candidate elastase inhibitory peptides exhibited robust inhibitory activity against elastase, but not cathepsin G, with z-API demonstrating the greatest potency (Fig. 3c, d).

**Table 1:** List of candidate inhibitory peptides and their protease targets

Peptide	Target
z-FLF-cmk	Cathepsin G
z-EPF-cmk	Cathepsin G
z-GLF-cmk	Cathepsin G
z-AFLF-cmk	Cathepsin G
z-GLW-cmk	Cathepsin G
z-GLK-cmk	Cathepsin G
z-KAL-cmk	IL-36α cleavage site
z-AAPV	Elastase
z-API	Elastase
z-APL	Elastase
z-APV	Elastase
z-ARPV	Elastase
z-RPV	Elastase
z-DTEF	IL-36β cleavage site
z-PQR	IL-36β cleavage site
z-RAV	IL-36γ cleavage site
z-RPI	Elastase
z-RPL	Elastase
z-RPV	Elastase
z-EPF-API-cmk	Cathepsin G/Elastase
z-API-EPF-cmk	Elastase/Cathepsin G

### Pseudosubstrates for elastase and cathepsin G can suppress IL-36 cytokine activation

**Fig. 4:**
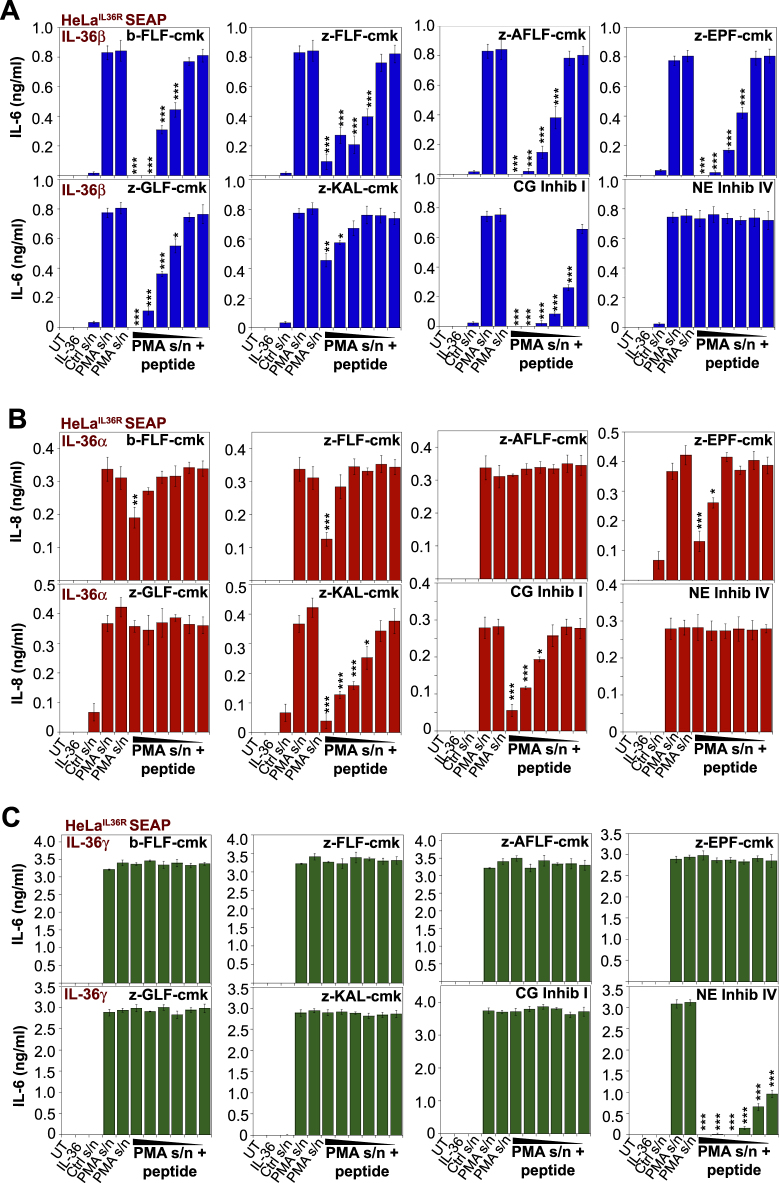
Novel peptide-based inhibitors of cathepsin G suppress IL-36β activation by proteases contained within neutrophil degranulates. **a**–**c** HeLa^IL-36R-SEAP^ cells were either left untreated, or were stimulated either with recombinant IL-36α, IL-36β, or IL-36γ (500 pM) that had been pre-incubated for 2 h at 37 °C, either alone, with unstimulated neutrophil supernatant (Ctrl s/n), or PMA-activated human neutrophil supernatant (PMA s/n) in the presence or absence of a titration (10, 5, 2.5, 1, 0.5, or 0.25 μM) of candidate peptide inhibitors. After 24 h, cytokine concentrations in the culture supernatants were determined by ELISA. Results shown are representative of at least three independent experiments. Error bars represent the mean ±SEM of triplicate determinations from a representative experiment. Asterisk(s) indicate significance levels, ***p < .0001, **p < .001, *p < .1, by Student’s *t* test

**Fig. 5:**
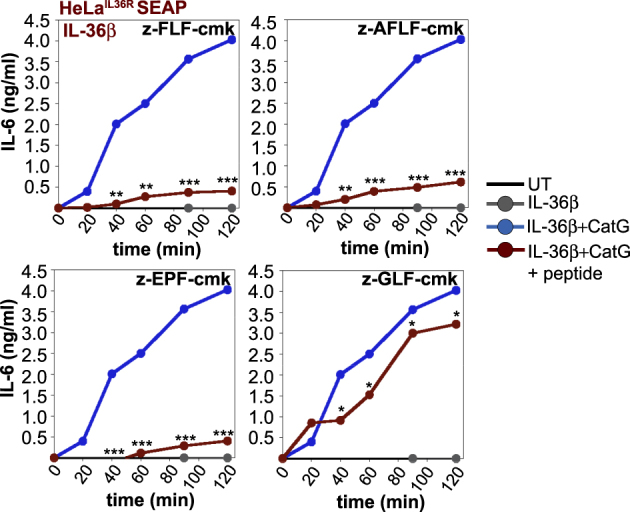
Time course analysis of inhibition of IL-36β activation by cathepsin G-specific peptide antagonists. Cathepsin G was pre-incubated for 30 min on ice in the presence or absence of the indicated cathepsin G peptide inhibitors (at 10 μM) followed by incubation at 37 °C with recombinant IL-36β (500 pM) for 20–120 min as indicated. HeLa^IL36R-SEAP^ cells were then incubated with samples of each reaction taken at the indicated time points. After 24 h, cytokine concentrations in cell culture supernatants were determined by ELISA. Results shown are representative of at least three independent experiments. Asterisk(s) indicate significance levels, ***p < .0001, **p < .001, *p < .1, by Student’s *t* test

Having found peptides that inhibited cathepsin G or elastase activity in the context of synthetic substrate peptide hydrolysis assays, we next explored the ability of these peptides to inhibit activation of all three IL-36 cytokines by neutrophil degranulate preparations, which contain a mixture of these proteases (Fig. 2b). To detect IL-36 cytokine activity, we used HeLa cells stably transfected with the IL-36 receptor^36^, as these cells secrete robust amounts of IL-6 and IL-8 in response to biologically active IL-36 (Fig. 1b, c). As Fig. 4a, b illustrate, activation of IL-36β by neutrophil degranulate preparations was robustly inhibited in the presence of the cathepsin G inhibitory peptides, z-FLF-cmk, z-AFLF-cmk, and z-EPF-cmk (Fig. 4a), with a modest effect of the same peptides on IL-36α activation (Fig. 4b). Once again we found that the inhibitory peptides were highly selective in their ability to antagonize activation of specific IL-36 cytokines, as cathepsin G-specific inhibitors failed to antagonize elastase-mediated IL-36γ activation by neutrophil degranulates, as expected (Fig. 4c). Time course analyses further confirmed the robust inhibitory effects of the same panel of inhibitors against IL-36β processing by cathepsin G (Fig. 5).

**Fig. 6:**
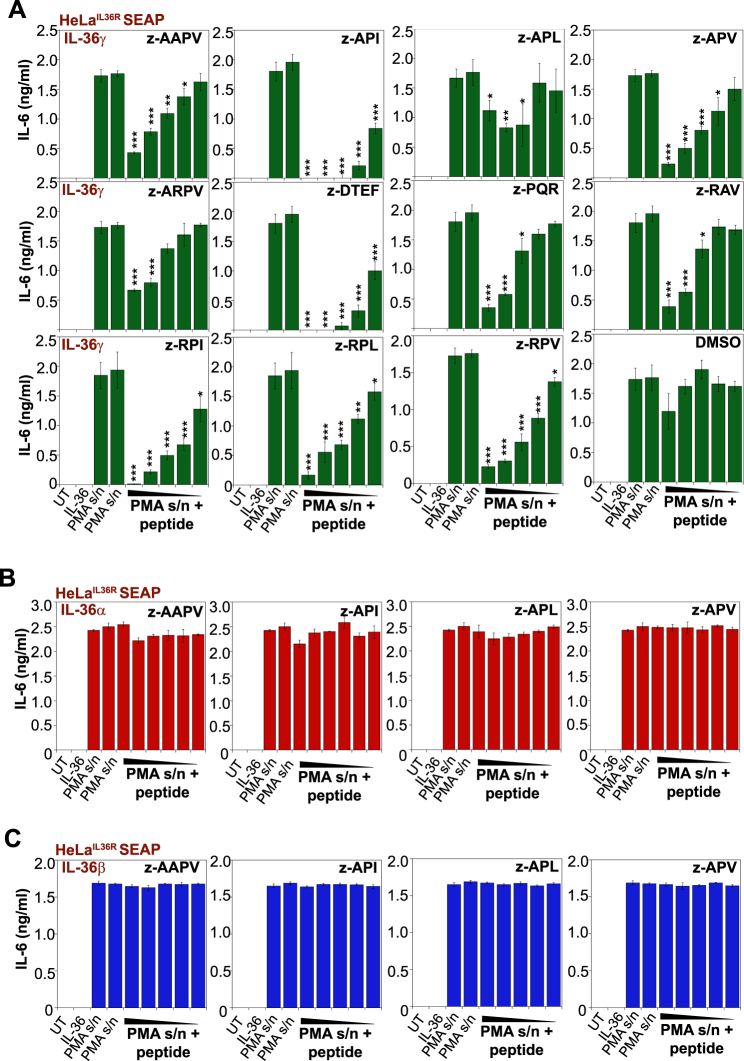
Novel peptide-based elastase inhibitors suppress IL-36γ activation by proteases contained within human neutrophil degranulates. **a**–**c** HeLa^IL-36R-SEAP^ cells were either left untreated, or were treated with recombinant IL-36α, IL-36β, or IL-36γ (500 pM) that had been pre-incubated for 2 h at 37 °C with PMA-activated human neutrophil degranulate, in the presence or absence of a titration (10, 5, 2.5, 1, 0.5, or 0.25 μM) of the indicated candidate peptide inhibitors. After 24 h, cytokine concentrations in the culture supernatants were determined by ELISA. Results shown are representative of at least three independent experiments. Error bars represent the mean ±SEM of triplicate determinations from a representative experiment. Asterisk(s) indicate significance levels, ***p < .0001, **p < .001, *p < .1, by Student’s *t* test

**Fig. 7:**
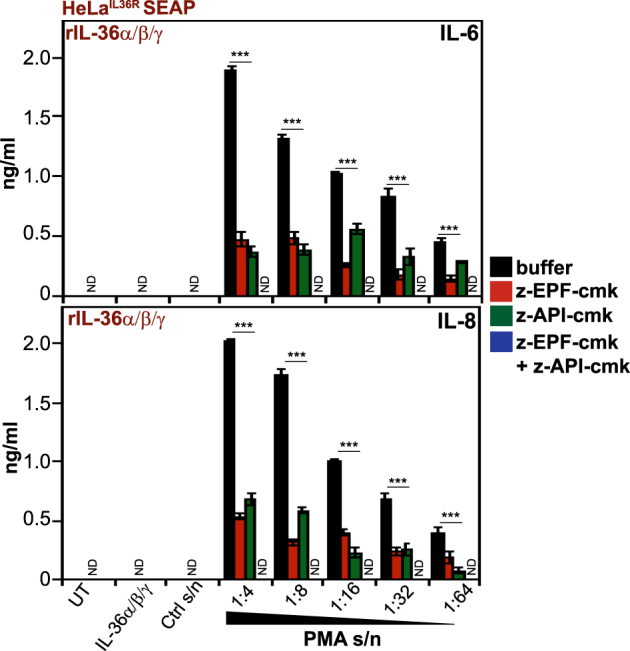
Combined inhibition of cathepsin G and elastase suppresses activation of all IL-36 subfamily cytokines by neutrophil proteases. HeLa^IL-36R-SEAP^ cells were stimulated with a combination of recombinant human IL-36 proteins (IL-36α + β + γ, all 500 pM final) pre-incubated for 2 h at 37 °C with a titration of PMA-activated human neutrophil degranulate, or control unstimulated neutrophil S/N 1:4, in the presence or absence of a fixed concentration (5 μM) of the best novel candidate cathepsin G (z-EPF-cmk) and elastase (z-API-cmk) peptide inhibitors either alone or in combination, as indicated. After 24 h, cytokine concentrations in the culture supernatants were determined by ELISA. Results shown are representative of at least three independent experiments. Error bars represent the mean ±SEM of triplicate determinations from a representative experiment. Asterisk(s) indicate significance levels, ***p < .0001, **p < .001, *p < .1, by Student’s *t* test. ND not detected, indicates where cytokine production was below the detection threshold of the assay

Similarly, the novel elastase-specific peptides (z-AAPV, z-API, and z-APV) antagonized activation of IL-36γ by the latter protease (Fig. 6a), with no effects on IL-36α or IL-36β activation by neutrophil degranulates (Fig. 6b, c). We then assessed the ability of the best performing cathepsin G (z-EPF-cmk) and elastase (z-API-cmk) inhibitors to suppress the activation of all three IL-36 family cytokines when these were simultaneously present. As illustrated in Fig. 7, the combination of all three IL-36 cytokines exhibited robust activity in the presence of PMA-activated neutrophil degranulates and this was significantly attenuated by either peptide inhibitor alone. However, the combination of elastase-specific and cathepsin G-specific inhibitors completely blocked the processing and activation of all three IL-36 cytokines when present concurrently (Fig. 7), as would be likely at many inflammatory sites.

### Bi-specific pseudosubstrates abrogate IL-36β and IL-36γ activation by neutrophil degranulates

**Fig. 8:**
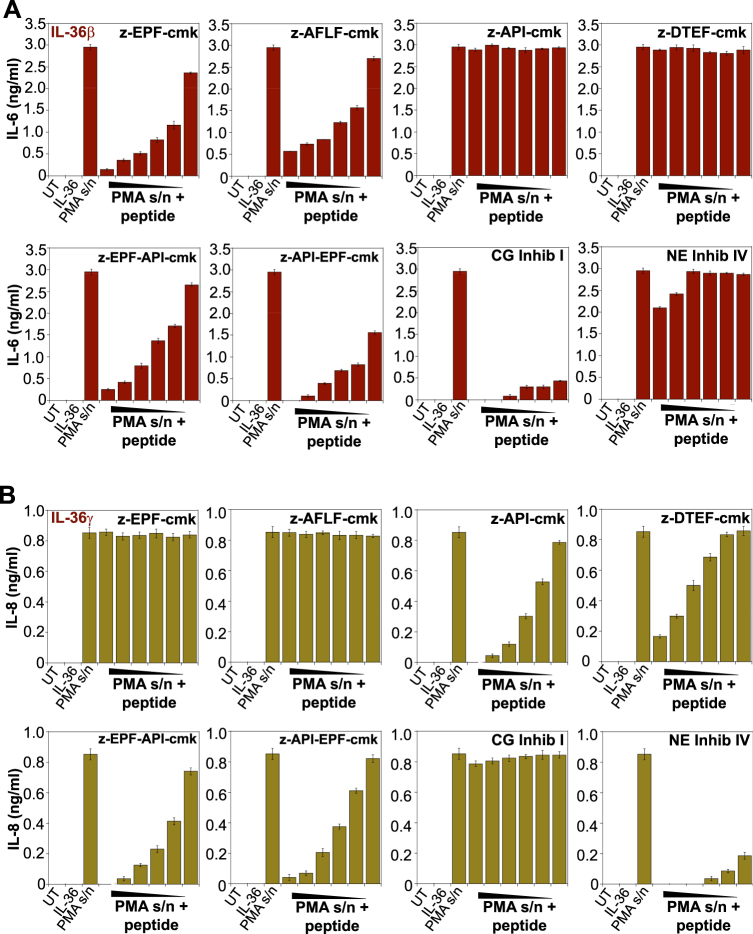
Bi-specific inhibitors of cathepsin G and elastase block IL-36β and IL-36γ activation. **a**,**b** HeLa^IL-36R-SEAP^ cells were stimulated with **a** IL-36β (500 pM) or **b** IL-36γ (500 pM), pre-incubated for 2 h at 37 °C with a fixed dose (1:4) of PMA-activated neutrophil degranulate (or control unstimulated neutrophil S/N) in the presence or absence of a titration (10, 5, 2.5, 1, 0.5, or 0.25 μM) of candidate peptide inhibitors. After 24 h, cytokine concentrations in the culture supernatants were determined by ELISA. Results shown are representative of at least three independent experiments. Error bars represent the mean ±SEM of triplicate determinations from a representative experiment

**Fig. 9:**
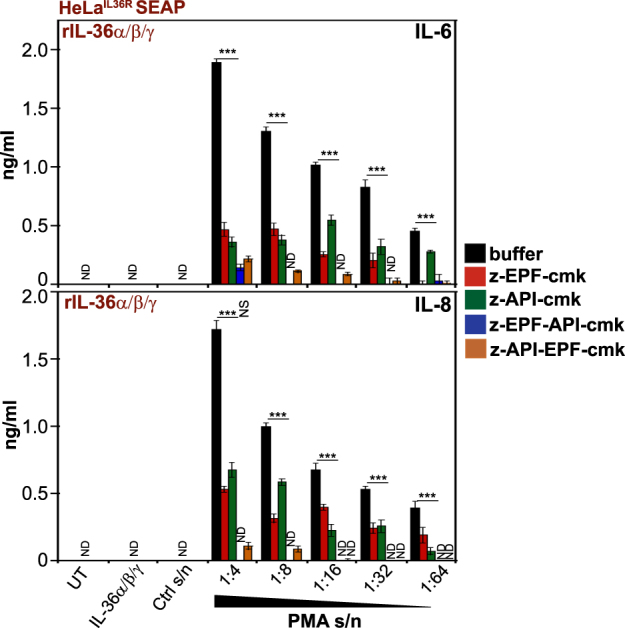
Bi-specific neutrophil protease inhibitors can suppress activation of all IL-36 subfamily cytokines simultaneously. HeLa^IL-36R-SEAP^ cells were stimulated with a combination of recombinant human IL-36 proteins (IL-36α + β + γ, all 500 pM final) pre-incubated for 2 h at 37 °C with a titration of PMA-activated neutrophil degranulate (or control unstimulated neutrophil S/N 1:4) in the presence or absence of the mono-specific (z-EPF-cmk or z-API-cmk) or bi-specific (z-EPF-API-cmk or z-API-EPF-cmk) cathepsin G/elastase peptide inhibitors (all at 5 μM). After 24 h, cytokine concentrations in the culture supernatants were determined by ELISA. Results shown are representative of at least three independent experiments. Error bars represent the mean ±SEM of triplicate determinations from a representative experiment. Asterisk(s) indicate significance levels, ***p < .0001, **p < .001, *p < .1, by Student’s *t* test. ND not detected, indicates where cytokine production was below the detection threshold of the assay

Having identified novel antagonists of cathepsin G and elastase activity, we next explored whether bi-specific peptide antagonists would also be effective at targeting both IL-36β and IL-36γ activation by activated neutrophil degranulates. Therefore, we generated the bi-specific peptides, z-API-EPF-cmk and z-EPF-API-cmk, and compared their ability to block neutrophil protease-mediated IL-36 processing. As demonstrated in Fig. 8, IL-36β activation by neutrophil degranulate was robustly inhibited by the cathepsin G peptide inhibitor (z-EPF-cmk), while IL-36γ activation was robustly inhibited by the novel elastase peptide inhibitor (z-API-cmk), as before. Furthermore, the bi-specific peptide antagonists z-EPF-API-cmk and z-API-EPF-cmk were also capable of suppressing both IL-36β and IL-36γ activation by PMA-treated neutrophil degranulates (Fig. 8). Similarly, when the combination of all three IL-36 cytokines was activated simultaneously through incubation with PMA-treated human neutrophil degranulates, the bi-specific peptides were also highly effective at suppressing IL-36 activation (Fig. 9). Thus, bi-specific cathepsin G and elastase peptide antagonists may be useful in the context of inflammatory reactions, where multiple proteases are present simultaneously.

### Processing of IL-36 cytokines by psoriatic skin eluates can be suppressed by pseudosubstrates for neutrophil proteases

**Fig. 10:**
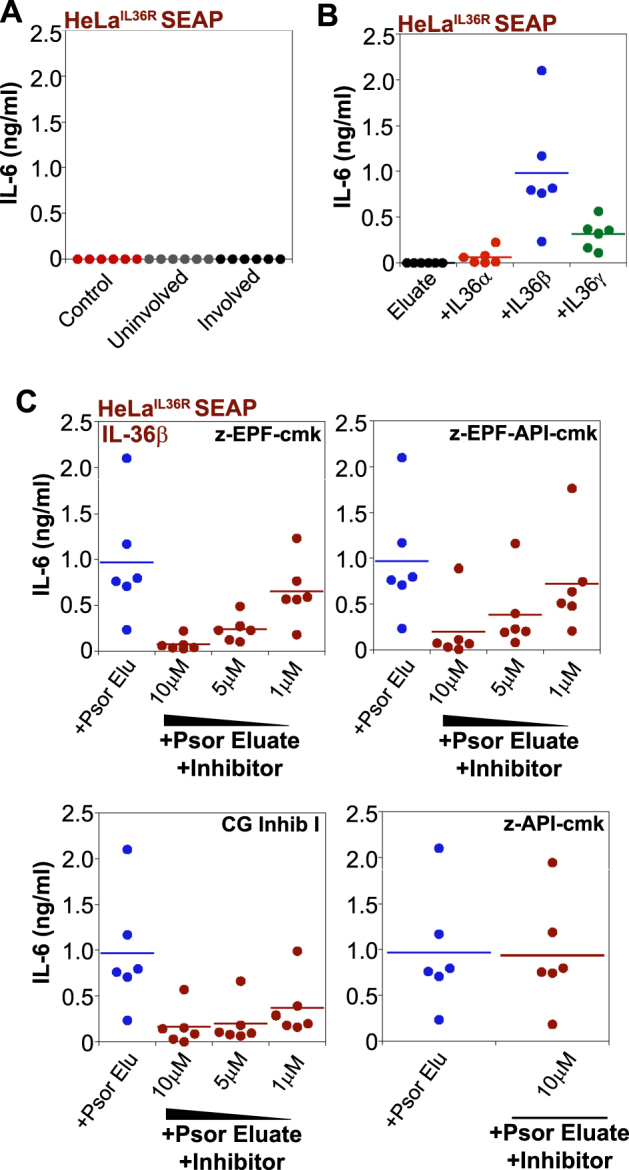
Psoriatic skin eluates contain IL-36β-processing activity that can be suppressed by cathepsin G-targeted pseudosubstrates. **a** HeLa^IL-36R-SEAP^ cells were stimulated with skin eluates from either healthy volunteer controls, or eluates from uninvolved (non-lesional) or involved (lesional) skin areas from psoriatic individuals (*n* = 6 per group). After 24 h, cytokine concentrations in the culture supernatants were determined by ELISA. **b** HeLa^IL-36R-SEAP^ cells were stimulated with skin eluates from lesional areas of psoriatic individuals (*n* = 6), either alone, or after incubation of the same eluates for 2 h at 37 °C with recombinant IL36α, -β, or -γ (500 pM). After 24 h, cytokine concentrations in the culture supernatants were determined by ELISA. **c** HeLa^IL-36R-SEAP^ cells were stimulated with IL36β (500 pM) that had been pre-incubated for 2 h at 37 °C with skin eluates from lesional areas of psoriatic individuals (*n* = 6), either alone (blue symbols), or in combination with the indicated concentrations of the novel cathepsin G (z-EPF-cmk) or cathepsin G/elastase (z-EPF-API-cmk) peptide inhibitors (maroon symbols). Cathepsin G Inhibitor I (Merck) served as a positive control and the elastase inhibitor (z-API-cmk) as a negative control (bottom panels). After 24 h, cytokine concentrations in the culture supernatants were determined by ELISA. Results shown are representative of at least three independent experiments

To explore the potential utility of neutrophil protease antagonists to suppress IL-36 cytokine activation in the skin, we used tape-stripped samples from affected skin areas of psoriatic individuals. Skin samples were incubated with CHAPs-containing buffer to facilitate elution of proteins. Our previous studies have shown that eluates from psoriatic individuals exhibit elevated levels of cathepsin G activity, as compared to healthy controls^14^. Consistent with this, we also found that psoriatic skin samples were capable of robustly processing and activating exogenously added IL-36β^14^. As can be seen from Fig. 10a, there was no detectable cytokine activity in these eluates when added directly to HeLa^IL-36R-SEAP^ cells due to the dilution factor of ~100-fold in these assays. However, exogenously added IL-36β (but not IL-36α or IL-36γ) was processed and activated upon addition to psoriatic skin eluates (Fig. 10b), consistent with our previous observations^12^. Because cathepsin G is the main IL-36β-processing protease (Fig. 1d and Ref. 12), this suggested that the cathepsin G inhibitory peptide, z-EPF-cmk, should suppress activation of the latter. As can be seen from Fig. 10c, this was indeed found to be the case, whereas the elastase-selective inhibitor z-API-cmk failed to have any effect on IL-36β-processing activity under the same conditions. Furthermore, the bi-specific peptide z-EPF-API-cmk also exhibited activity in this assay (Fig. 10c, top right), as did a small molecule inhibitor of cathepsin G (Fig. 10c, bottom left).

Collectively, our data identify novel peptide-based inhibitors of cathepsin G or elastase and demonstrate proof-of-principle that such inhibitors might have potential as antagonists of IL-36 cytokine activation in disease settings, such as psoriasis, that are associated with elevated IL-36 cytokine activity. Such inhibitors have the advantage that they can be applied directly to active skin plaques, rather than systemically, thereby avoiding unwanted global suppression of neutrophil protease activity or IL-36 cytokine activation in other locations.

## Discussion

Here, we report the identification of novel mono-specific and bi-specific peptides, which exhibit significant inhibitory activity against elastase or cathepsin G, that can antagonize the processing and activation of all IL-36 family cytokines by the latter proteases. Because elastase and cathepsin G have also been implicated in the processing and activation of IL-1α, IL-1β, IL-33, IL-36RA, and other cytokines^37–41^, the peptides reported herein may also suppress the activation of multiple members of the extended IL-1 family.

IL-1 family cytokines are central players in sterile inflammatory diseases and autoinflammatory disorders including rheumatoid arthritis, psoriasis, gout, neurodegenerative disorders, and atherosclerosis, as well as systemic autoinflammatory diseases such as cryopyrin-associated periodic syndromes (CAPS)^2^. Thus, targeting of proteases responsible for the activation of multiple IL-1 family members represents an attractive therapeutic strategy. Given the apical nature of IL-36 cytokine signaling in inflammatory cascades and the relatively confined expression pattern of IL-36 cytokines to skin and barrier tissues, significant therapeutic improvement may be achievable through targeting this cytokine subfamily, with less of the systemic side effects associated with treatments targeting other apical cytokines, such as TNFα.

There is now a plethora of genetic, in vitro and in vivo evidence implicating IL-36 cytokines in the development of inflammatory skin conditions, most notably psoriasis. For example, transgenic overexpression of IL-36α in the mouse leads to a psoriasis-like condition at birth and that can be further exacerbated with the skin irritant, phorbol acetate^14,15^. The most severe form of psoriasis (GPP) has recently been shown to be an inherited disorder associated with hypomorphic mutations (partial loss-of-function) in IL-36RA, the endogenous countermeasure to excessive IL-36 activity^17,18,21^. The observation that a modest reduction in the function of IL-36RA is sufficient to promote a life-threatening form of psoriasis is indicative of the central role for IL-36 signaling in the skin. Further studies have shown that application of a TLR7 agonist (Imiquimod) to the skin of humans and mice can initiate psoriatic lesions that are dramatically worsened on an IL-36RA null background, whereas imiquimod-induced psoriasis is completely abolished in IL-36R-deficient mice^19,25^. IL-36 proteins are highly expressed in keratinocytes and upregulated in response to IL-17, IL-22, and TNF– cytokines that are frequently overexpressed in psoriasis^16,42,43^. Related to this, it has recently been shown that IL-36 cytokines, in particular IL-36β, can induce robust production of pro-inflammatory cytokines (such as IL-6 and TNF) from diverse cell types including skin resident (Langerhans) DCs and macrophages, as well as keratinocytes^10^.

A characteristic feature of psoriatic inflammation is robust neutrophil-rich cellular infiltrates^26,27,44^. Neutrophils play a critical role in the first phase of the immune response to infection or tissue damage^45–47^. While release of neutrophil proteases can exert important protective effects during infection, if left unchecked these proteases can cause collateral tissue damage and amplify inflammation^48–50^. Thus, damage to barrier tissues, as a result of infection or tissue trauma, that results in neutrophil recruitment, release of neutrophil-derived proteases and subsequent processing and activation of IL-36 cytokines, may play an important initiating role in psoriasis. This is particularly relevant in the context of individuals lacking endogenous buffers of IL-36 activity, such as deficiency in the IL-36R antagonist (DITRA)^17,18,21–23^. Although neutrophil protease inhibitors have not yet been approved for use in the treatment of psoriasis, targeted depletion of neutrophils in patients with GPP led to significant clinical improvement, providing support for the rationale for targeting neutrophil proteases in the context of skin inflammation^28^.

At present, biologics directed against the cytokines TNFα, IL-17, IL-22, and IL-12/23 have been approved for the treatment of moderate-severe plaque psoriasis by the FDA and EMA^31^. However, while this approach can be effective, systemic administration of cytokine-neutralizing antibodies can have serious negative side effects including increased susceptibility to opportunistic infections, re-activation of hepatitis B/tuberculosis (TB), and risk of development of lymphoma^51,52^. Indeed, a recent meta-analysis revealed that biologics are associated with significantly higher rates of adverse events in the short term, precluding their use in less severe cases^30^. Furthermore, systemic administration of these therapies may fail to reach sufficient plasma concentrations to be effective, suffering the effects of first pass metabolism. In contrast, topical application of therapeutic peptides or small molecule protease inhibitors to affected mucosa may be of particular benefit in skin inflammatory conditions, owing to their ease of application, lower cost, localized effects, and patient familiarity with existing topical treatments for their condition.

The primary challenges associated with peptide delivery include overcoming difficulties with skin penetration and maintaining peptide stability^53^. It is generally regarded that molecules >400 Da are unsuitable for transdermal delivery^54^. However, in psoriasis patients it has been demonstrated that measures of transdermal molecular trafficking are increased^55,56^, thus the >400 Da cutoff may not strictly apply in this context. Furthermore, a variety of skin penetration techniques exist to overcome this obstacle including chemical techniques (e.g., permeation enhancers such as alcohols, amines, surfactants, esters, lipid-systems, or pro-drug formulations) and physical techniques (e.g., iontophoresis, electroporation, and microporation). Similarly, techniques to improve peptide stability have also been developed to overcome this limitation and include direct structural modification (e.g., cyclization and PEGylation), co-administration with enzyme inhibitors, hyperglycosylation and use of carrier systems (e.g., liposomes, micelles, and nanoparticle carriers). Thus, viable topical peptide antagonist delivery systems are currently available.

As an alternative to peptide-based antagonists of neutrophil proteases, a number of small molecule inhibitors of neutrophil cathepsin G or elastase/proteinase-3 have been reported previously^35,57–61^. Although clinical trials have been conducted with these compounds to explore their potential utility in acute lung injury and infection-driven pulmonary inflammation, to our knowledge, none of these inhibitors have been explored for their potential to suppress inflammation in the skin. Although the latter inhibitors have demonstrated limited efficacy in lung models of infection or injury-driven lung inflammation, it may be worth exploring their impact on psoriatic inflammation in future studies. Potential limiting factors relating to the failure of small molecule neutrophil protease inhibitors to demonstrate utility in pulmonary models of inflammation to date, may relate to issues of poor bioavailability, poor tissue penetrance, and lack of selectivity in vivo. However, direct application of such inhibitors to affected areas of inflamed skin, rather than via oral or intravenous administration, may represent a promising approach for future investigations.

In summary, here we have identified several novel peptide-based inhibitors of neutrophil-derived cathepsin G or elastase that may have potential as therapeutic modulators of IL-36 cytokine activity in inflammatory conditions such as psoriasis.

## Experimental procedures

### Reagents

Synthetic peptides, Ac-DEVD-AMC and suc-FLF-sBzl, were purchased from Bachem (Germany); Suc(oMe)-AAPV-AMC was purchased from Peptanova (Germany). Synthetic peptides, biotin-FLF-cmk, z-FLF-cmk, z-EPF-cmk, z-AFLF-cmk, z-GLF-cmk, z-GLW-cmk, z-GLK-cmk, z-AAPV, z-API, z-APL, z-APV, z-ARPV, z-RPI, z-RPL, z-RPV, z-PQR, z-DTEF, z-API-EPF-cmk, z-EPF-API-cmk, and -cmk derivatives thereof were synthesized by Boston Open Labs (USA). Chemical inhibitors Cathepsin G Inhibitor I (219372) and Elastase Inhibitor IV (324759) were purchased from Calbiochem (UK). Purified neutrophil-derived cathepsin G was purchased from Calbiochem (UK). Purified neutrophil-derived elastase was purchased from Serva (Germany). Unless otherwise indicated, all other reagents were purchased from Sigma (Ireland) Ltd.

### Cell culture

HeLa cells were cultured in RPMI media (Gibco), supplemented with 5% fetal calf serum (FCS). HeLa.vector or HeLa.IL-36R cell lines were generated by transfection with pCXN2.empty or pCXN2.IL-1Rrp2 (IL-36R) plasmids followed by selection using G-418 antibiotic (Sigma). IL-36R over-expressing clones were expanded from single cells. Clones were selected by detection of responsiveness to active forms of IL-36 via ELISA. The HeLa.IL-36R.SEAP cell line was generated by transfection with pNifty2-SEAP plasmid (InvivoGen) followed by selection using zeocin antibiotic, as previously described^36^. Clones were expanded from single cells and tested for SEAP production. All cells were cultured at 37 °C in a humidified atmosphere with 5% CO_2_.

### Expression and purification of recombinant IL-36 and caspases

Full-length IL-36α, IL-36β, and IL-36γ cytokines were generated by cloning the human coding sequences in frame with the poly-histidine tag sequence in the bacterial expression vector pET45b. Protein was expressed by addition of 600 μM IPTG to exponentially growing cultures of BL21 RIL strain *E. coli* followed by incubation for 3 h at 37 °C. Bacteria were lysed by sonication and poly-histidine-tagged proteins were captured using nickel-NTA agarose (Qiagen, UK), followed by elution into PBS, pH 7.2, in the presence of 100 mM imidazole. Modified forms of IL-36 were also generated that included an N-terminal caspase-3-processing motif (DEVD) in the IL-36 coding sequences^36^. Recombinant poly-histidine-tagged caspases -1 and -3 were also expressed and purified as described previously^62^.

### Protease activity assays

Reactions (50 μl final volume) were carried out in protease reaction buffer (50 mM HEPES, pH 7.4, 75 mM NaCl, 0.1% CHAPS, 2 mM DTT) containing 50 μM Ac-DEVD-AMC, or Suc(oMe)-AAPV-AMC. Samples were measured using an automated fluorimeter (SPARK 10 M; TECAN) at wavelengths of 430 nm (excitation) and 535 nm (emission). For suc-FLF-sBzl assay, substrate was diluted to a final concentration of 300 μM in protease reaction buffer (50 mM HEPES, pH 7.4, 75 mM NaCl, 0.1% CHAPS, DTNB 300 μM). Cathepsin G hydrolyzes the synthetic substrate suc-FLF-sBzl with the release of the thiobenzyl group. The free thiobenzyl group reacts with DTNB [5,5′-dithiobis(2 nitrobenzoic acid) and produces a chromophore (TNB), which absorbs at 430 nm. Samples were measured by automated fluorimeter (SPARK 10 M; TECAN).

### Protease cleavage assays

Reactions (40–100 μl, final volume) were carried out in protease reaction buffer (50 mM HEPES [pH 7.2], 75 mM NaCl, and 0.1% CHAPS) for 2 h at 37 °C. For IL-36 bioassays, IL-36 cytokines were typically cleaved at a 50 nM concentration and subsequently diluted onto target cells at a final concentration ranging from 0.25 to 1 nM.

### Measurement of cytokines and chemokines

Cytokines and chemokines were measured from cell culture supernatants using specific ELISA kits obtained from R&D systems (human IL-6 and human IL-8). All cytokine assays were carried out using triplicate samples from each culture.

### Tape strip samples from control and psoriatic skin

Healthy control volunteers (*n* = 6) and patients with mild/moderate psoriasis (*n* = 6) and not receiving treatment in the previous 6 months were recruited. Fixomull (2 × 2 cm) adhesive tape strips were applied to healthy control, uninvolved or involved psoriatic skin under firm pressure for 10 s. The tape strips were removed gently, placed in sterile 1.5 ml Eppendorf tubes, and eluted with protease reaction buffer (PRB) (50 mM HEPES [pH 7.2]/75 mM NaCl/0.1% CHAPS) under constant rotation for 1 h at 4 °C. Skin eluates were stored at −80 °C. Bio-activity assays were conducted according to the protease cleavage assays outlined above.
